# Factors Related To The Loneliness Of Older Women With Hypertension In Tehran

**DOI:** 10.1093/geroni/igab046.2326

**Published:** 2021-12-17

**Authors:** Ali Darvishpoor Kakhki, Sona Mohammadi, Reza Miri

**Affiliations:** Shahid Beheshti University of Medical Sciences, Tehran, Iran

## Abstract

Older women have longevity and face with common chronic diseases such as hypertension longer than men. In addition the refusal to accept older women into the mainstream of society can affect the loneliness of older women particularly in developing countries such as Iran. This study was conducted to describe factors related to loneliness of older women with hypertension in Tehran. This descriptive, correlational study was conducted on a sample of 300 older women above age 60 in five regions of Tehran in 2020. A socio-demographic questionnaire and the Russell Loneliness Scale were used for data collection. Content validity and Cronbach’s alpha were used for evaluating the validity and reliability of questionnaires. 61% of older women were widowed and 37.3% lived alone with a mean age of 72.16(± 8.5) year. The mean score for loneliness was 66.26 (±13.44) on a 20 to 80-point scale. The scores of loneliness were influenced significantly by not having an income source, no living companion, chronic diseases, hospitalization in last year, family history of hypertension, and duration of hypertension. The best predictors of loneliness were hospitalization in last year, duration of hypertension, family history of hypertension, and chronic diseases. The findings of this study showed that loneliness is very common in older women with hypertension and is related to a number of factors. Monitoring modifiable factors such as hospitalization in the last year and non-modifiable factors such as duration of hypertension will help us to prevent or reduce loneliness in older women with hypertension.

